# Trace Element Concentrations of Arsenic and Selenium in Toenails and Risk of Prostate Cancer among Pesticide Applicators

**DOI:** 10.3390/curroncol31090405

**Published:** 2024-09-14

**Authors:** Leslie K. Dennis, Marvin E. Langston, Laura Beane Freeman, Robert A. Canales, Charles F. Lynch

**Affiliations:** 1 Department of Epidemiology & Biostatistics, Mel and Enid Zuckerman College of Public Health, University of Arizona, Tucson, AZ 85721, USA; marvin.langston@stanford.edu; 2 Department of Epidemiology, College of Public Health, University of Iowa, Iowa City, IA 52242, USA; charles-lynch@uiowa.edu; 3 Department of Epidemiology and Population Health, Stanford University School of Medicine, Stanford University, Stanford, CA 94305, USA; 4 Division of Cancer Epidemiology and Genetics, National Cancer Institute, Rockville, MD 20850, USA; freemala@mail.nih.gov; 5 Department of Environmental and Occupational Health, Milken Institute School of Public Health, George Washington University, Washington, DC 20052, USA; rcanales@gwu.edu; 6 Department of Community and Environmental Health, Mel and Enid Zuckerman College of Public Health, University of Arizona, Tucson, AZ 85721, USA

**Keywords:** trace elements, biomarkers, arsenic, selenium, prostate cancer

## Abstract

Prostate cancer is a common cancer among males in the US, but little is known about its risk factors, including trace elements. The primary aim of this study was to examine prostate cancer and its association with arsenic and selenium in toenails. We conducted a small, nested case-control study of men residing in Iowa within the Agricultural Health Study cohort, where we also collected toenail samples to test for arsenic and other trace elements. Toenail samples were sent for neutron activation analysis aimed at long-lived trace elements, including arsenic. Logistic regression was used to estimate odds ratios (ORs) for trace element exposures and prostate cancer. A total of 66 prostate cancer cases and 173 healthy controls returned questionnaires, over 99% of which included toenail samples. An increased risk was seen for the highest levels of arsenic (OR = 3.4 confidence interval (CI) of 1.3–8.6 and OR = 2.2, 95% CI of 0.9–5.6) and the highest level of selenium (2.0, 95% CI of 1.0–4.0). These data also show detectable levels of over 50% for 14 of 22 elements detected in the toenails. The association seen here with arsenic and prostate cancer further supports ecological studies finding an association with community levels of arsenic and prostate cancer incidence and mortality.

## 1. Introduction

Prostate cancer has the highest incidence rate among cancers in men in the United States, excluding non-melanoma skin cancers [1]. Few risk factors for prostate cancer have been identified. Known risk factors include benign prostatic hyperplasia [2], family history, and African-American race [3]. Little is known about modifiable risk factors for prostate cancer other than a low-lycopene diet [4]. Possible associations include arsenic [5,6], selenium [7,8,9,10,11,12], obesity [13], animal-based fat intake [14,15], cooking carcinogens [16], and occupational exposures. Occupational studies among farmers have suggested several chemical and pesticide exposures may increase risk [17,18,19,20,21,22,23,24,25]. Further exploration of environmental factors among pesticide applicators, primarily farmers, can shed light on risk factors that may help explain the increased prostate cancer risk among farmers. 

Several trace elements are associated with an increased risk of cancer, including arsenic, while selenium is often associated with a decreased risk [26], but few have been extensively considered in population-based studies for prostate cancer. In in vitro and cell culture studies, trace levels of arsenic have been shown to transform human prostate epithelial cells [27] and to alter the response of stromal cells to tumor cells, thereby enhancing their potential for growth [28]. Selenium is an antioxidant and has been shown to reduce free-radical formation [29], thereby protecting against DNA damage attributed to oxidative stress. Measurement of individual exposure to these trace elements can be complex. Trace element intake derived from food frequency questionnaires is likely to have large amounts of misclassification due to difficulty with recall and may not account for other routes or pathways of exposure (e.g., inhalation or water intake). Biomarkers of trace elements, including arsenic, can be assessed from blood, adipose tissue, urine, hair, and nails. Of these, toenails are the least invasive biospecimen to collect that represents exposure 9-12 months prior to collection [30].

We conducted a small case-control study of prostate cancer, nested within the Agricultural Health Study (AHS) cohort, to examine the association with several trace elements, with specific interest in arsenic and selenium. Toenail samples were collected from men with prostate cancer and healthy controls. 

## 2. Materials and Methods

### 2.1. Study Population

The AHS is an on-going cohort study of mostly male farmers examining the relationship between agricultural exposures and disease in pesticide applicators in Iowa and North Carolina. The AHS in Iowa includes 36,215 certified pesticide applicators who are male farmers. The Iowa Cancer Registry (ICR), part of the national Surveillance, Epidemiology, and End Results (SEER) Program of the National Cancer Institute, tracks the cancer experience of cohort members through periodic electronic linkage. We conducted a nested case-control study of prostate cancer within Iowa participants of the AHS cohort (data release version 021304) to examine trace elements found in toenails. The prostate cases were males, age 18 years or older at diagnosis, diagnosed from July 2003 through December 2004 (surveyed December 2003 through August 2005), residents of the state of Iowa, AHS participants, able to speak English, and diagnosed with histologically confirmed prostate cancer. Prostate cancer cases were identified from pathology reports as standard practice by the ICR/SEER using a semi-rapid reporting method in an attempt to obtain cases within 12 months of diagnosis so that the toenails would refer to exposures prior to diagnosis. Controls were randomly selected among healthy male AHS participants who had not been contacted in the prior month and did not have cancer. The controls were also males, age 18 years or older, able to speak English, residents of Iowa, and in the AHS cohort. They were frequency-matched to cases at a 3:1 ratio based on age. The cohort of pesticide applicators (farmers) had little ethnic/racial variation. The Supplementary Materials shows Figure S1 that describes the recruitment.

### 2.2. Survey of Subjects

Each participant was mailed a questionnaire and instructions for submitting a sample of their toenail clippings for analysis. The median weight of the toenail samples was 157 mg (range 72 to 586 mg). The case-control survey included information on fruit and vegetable consumption, supplementation, known exposure to arsenic, cadmium, or Brom-O-Gas (or Brom-O-Sol or methyl bromide), PSA testing, vasectomy, and several medical conditions. The case-control survey more specifically included information asked about years in their current residence, primary water source for drinking and cooking water, prior residence if <1 year in current home, fruit and vegetable consumption, where tomatoes and vegetables came from, self-reported known exposures to arsenic, cadmium, and Brom-O-Gas/Brom-O-Sol, having a prostate-specific antigen test in last 2 years, ever having certain medical conditions, including infections, supplement use in the past year, and current marital status. Other information was obtained from baseline cohort questionnaires (Phase I), including smoking, height, and weight (to calculate BMI), along with self-reported known exposures to arsenic, herbicides, fungicides, fumigants, other potentially harmful exposures from farming and pesticide application, and family history of prostate cancer. The human subjects’ approval was received from the University of Iowa’s Institutional Review Board. This study was performed in accordance with the Declaration of Helsinki. 

### 2.3. Trace Element Analyses

Neutron Activation Analysis (NAA), a sensitive analytical technique, was used to measure the long-lived indicators of trace elements in the toenails. These analyses were conducted in 2006 at the facilities at Pennsylvania State University. For many elements, NAA offers sensitivities that are superior to those attainable by other methods, on the order of parts per billion or better. NAA is both accurate and reliable in measuring multiple long-lived indicators of trace elements in toenails [30]. Results were provided in parts per million (PPM). The limit of detection (LOD) is different for each element in each sample, related to the weight of the sample and relative abundance of each element. Non-detectable levels of arsenic (AS) in the toenails had LODs ranging from 0.02 to 1.7 PPM for 126 subjects (53%), while non-detectable levels of selenium (SE) had LODs ranging from 0.1 to 1.1 PPM among 68 subjects (28%). 

### 2.4. Limit of Detection (LOD)

The limit of detection varied by the toenail weight of the sample provided. A variety of transformation methods have been proposed for left-censored data below the LOD, including no change, dividing the detection limit by 2 or by the square root of 2, and imputation using maximum likelihood estimation (MLE). When looking at different percentages of censored values, Craghan & Egeghy [31] compared the LOD transformations of adding 0 (no change) and dividing the detection limit by 2 or by the square root of 2. They found the better method depended on the percent of censored values; the LOD divided by the square root of 2 provided the best estimates at both 25% below the LOD with 0% error and at 50% below the LOD but with potentially 33% error [31]. Similarly, Canales et al., [32] compared estimation methods including substitution of LOD/(square root of 2), along with MLE and Kaplan–Meier methods and two multiple imputation methods (one using MLE assuming a lognormal distribution and the second assuming a uniform distribution). Using data sets with known censoring, they compared his methods of finding means and standard deviations for the LOD/(square root of 2) for medium (35%) and high (65%) censoring that were similar to the known data, as were those for the multiple imputation method using MLE for a lognormal distribution [32]. Therefore, we used two methods of value imputation for trace element concentrations below the LOD were imputed. The first method, LOD/square root (2), has been shown to produce estimates with smaller bias and error rates for both levels of missing (for AS and SE) than other simple replacement techniques [31,32]. Additionally, a robust imputation or “fill-in” method was implemented that relies on assuming that all concentration values, including those that fall below limits of detection, follow a common distribution [33]. Using the fitdistcens function within the R package fitdistRplus, lognormal parameters defining this common distribution were estimated with MLE. This modified MLE method incorporates uncensored values, the number of censored observations, and the limits of detection for individual measurements. Random draws from the defined distribution were then used to “fill-in” censored values, bound by the limits of detection. In simulations comparing imputation and estimation techniques, this robust MLE method was found to be accurate for environmental applications in water quality investigations [33] and microbial risk assessments [32]. After the missing value imputation was completed, the data were transformed using the natural logarithm, and thereafter transformed back to PPM for reporting purposes.

Arsenic and selenium were divided into quartiles based on all subjects with data above the LOD, then the bottom two quartiles were combined to create the stronger reference category. When those below the LOD were estimated and added to the analyses, this changed to the distribution shown in the results tables.

### 2.5. Statistical Methods

The range of detectable levels for each trace element are reported. Per the protocol, seven subjects who turned their toenail samples in more than 12 months after diagnosis were excluded, as their toenail samples would not reflect exposures prior to cancer development. As the outcome is dichotomous and the dependent variable is ordinal, and no collinearity was seen, the assumptions of logistic regression have been met. Logistic regression was used to estimate odds ratios for exposures and prostate cancer. For arsenic and selenium, we looked at three exposure levels in an attempt to see if there was a dose–response relationship. Odds ratios (OR) and 95% confidence intervals (CI) are reported. Since prostate issues could be related to diagnosis of cancer, they were not considered as confounders. Confounding was examined for other potential risk factors (BMI, vegetable consumption related to lycopene) marginally associated with prostate cancer (*p* < 0.30), along with age (to account for residual confounding after frequency matching on age). 

## 3. Results

The participation rate for returning the mailed questionnaire and toenails among Iowa SEER cases was 63% and 57% among controls (only two controls returned the questionnaire without the toenails; <1%), as seen in Figure S1. Eighty-five percent of cases were recruited within 9 months of diagnosis. Participants were older and more educated (non-significantly) than nonparticipants (Table S1). They were not different by race (no variation in this Iowa population), Hispanic origin, vegetables consumption, ever smoking (100 cigarettes), or alcohol consumption in the past year (Table S1). Seven cases sent toenails samples after 12 months; five cases and two controls sent toenail samples that were insufficient (<3%) to examine in NAA analyses (23–69 mg), leaving 59 cases and 173 controls, ages 44–89 years, with toenail samples. The average age at diagnosis was 65; the average body-mass index was 28. While 45% of our subjects had smoked 100 or more cigarettes in their lifetime, 5–6% reported being former smokers. The average number of cigarette pack-years among current smokers was 31.1 and was 13.4 among former smokers; however, smoking rates were low.

Most lifestyle factors were similar between the prostate cancer cases and the healthy male controls. No differences were seen by age category, education, body-mass index, family history of prostate cancer, or smoking status (Table 1). Marginal differences were seen for marital status (5% more cases were married) and smoking pack-years (cases had higher pack-years even though a smaller percentage were ever smokers). Differences were not generally seen for the items on the list of medical conditions, including sexually transmitted infections, for which the prevalence was low in this population. There were associations, however, for enlarged prostate of 4.8 and non-significant increased risks for prostatitis (OR = 1.9) (Table 2). While enlarged prostate and prostatitis are related to prostate cancer in these data, these tend to be markers of prostate cancer and therefore were not controlled for as potential confounders. Based on the baseline cohort questionnaire, other factors related to farming, including ever applying broad categories of pesticides (herbicides, fungicides, and fumigants), along with exposure to solvents, wood dust, mineral dust, lead, and mercury, did not show differences between cases and controls. Table 2 also reports arsenical pesticides. Self-reported exposures, from the baseline cohort questionnaire (Phase I), of lead arsenate and lead poisoning showed increased, non-significant odds ratios (>2.0) suggesting possible associations but were based on less than 10 exposed subjects. 

The detectable levels of arsenic in the toenails ranged from 0.10 to 1.80 PPM, while detectable levels of selenium ranged from 0.12 to 2.53 PPM. Table 3 reports an association between prostate cancer and arsenic exposure with ORs for the upper two quartiles compared to the bottom two quartiles adjusted for age and pack-years of smoking. A clear dose–response relationship was not seen in these data (Table 3). Non-significant, negative, and positive associations were found for the top two quartiles of selenium exposure and prostate cancer.

A secondary aim was to describe what other trace elements were found when analyses were focused on arsenic. Selenium was detected in 72% of toenail samples. Aluminum, bromine, chlorine, sodium, and zinc were detected in 100% of the toenail samples. Additionally, cobalt, gold, magnesium, manganese, and potassium were detected in 75–99% of subjects, while antimony, calcium, chromium, copper, lithium, and vanadium were detected in 43–72% of samples, with iodine, iron, mercury, and scandium in only 24–37%. These are shown in Table 4, which includes the seven cases who turned in toenails after 12 months. Table 4 shows detectable levels of over 50% for 14 of 22 elements detected in the toenails.

## 4. Discussion

Our case-control study among male farmers in Iowa found an increased risk of prostate cancer with arsenic levels in toenails, similar to another Iowa study finding increased prostate cancer with low-level drinking water arsenic [34]. Selenium did not appear to have any significant association with prostate cancer in this study. The two different methods for handling samples with arsenic and selenium below the levels of detection showed similar trends. Ecological data have suggested an association between high arsenic levels and prostate cancer, although, to our knowledge, there are no case-control studies that have examined associations between arsenic and prostate cancer incidence. One cohort study of American Indians reported a 4-fold increase in prostate cancer mortality when comparing the highest baseline category of urinary arsenic concentration to the lowest [35], but they did not look at incidence.

Historically, the association between arsenic exposure and prostate cancer was first explored through ecological mortality studies mainly in Taiwan. An early study in Taiwan found a positive association between arsenic concentration in drinking water and community-level prostate cancer mortality in high arsenic endemic areas [36,37]. Ecological studies in low endemic areas, Spain and the US, have also found positive associations with prostate cancer mortality. In Spain, a study of prostate cancer mortality and arsenic in topsoil found a positive association [38]. A United States study of residential drinking water in Utah found increased prostate cancer mortality (standardized mortality ratio of 1.45: 95% CI = 1.07–1.91) for death among those exposed to medium to high levels of arsenic [39], whereas low-level arsenic exposure from drinking water was found to be associated with prostate cancer in Iowa [34]. These ecologic studies have been supported by more recent analytic studies of arsenic exposure and prostate cancer. These studies provide evidence for an association with mortality in both the high endemic arsenic areas of Taiwan and relatively low endemic arsenic areas in the United States. Most of these studies cannot provide much insight on causality due to the lack of temporal relationships, but the dose–response relationships seen for arsenic levels and prostate cancer mortality [35,36,38,39] strengthen the argument for causal claims.

Ecological studies have conflicting reports regarding prostate cancer incidence and arsenic in drinking water. In a high endemic arsenic region of Australia, areas with increasing concentrations of arsenic in water and/or soil had an increased standardized incidence ratio for prostate cancer [40]. A study in Argentina, a high endemic arsenic location, found an increased risk of prostate cancer (OR = 3.55: 95% CI 1.59–7.94) comparing those who lived in areas with drinking water concentrations above 10 µg/L to those who lived in areas with average concentrations below this level [41]. Another study in the same area of Argentina [42] reported a decreased incidence of prostate cancer with arsenic concentration in drinking water that, when categorized, showed no association. Studies reporting on arsenic exposure in drinking water and prostate cancer incidence in low endemic arsenic areas have shown conflicting results, with no association in a Denmark-based study [43] and a 10% increased risk for an Illinois-based ecological study [44].

This case-control study allowed for the investigation of the possible magnitude of association between a biomarker of arsenic exposure (toenails) and prostate cancer. Similar to other case-control studies of prostate cancer, having an enlarged prostate was associated with prostate cancer (OR = 4.8). To our knowledge, this is the first study to estimate arsenic concentration in toenails in relation to prostate cancer. More etiologic research is needed to determine if arsenic exposure (low, high, or both) is associated with the development of prostate cancer.

While we did not see an association with selenium and prostate cancer, a meta-analysis of 25 studies concluded that selenium seems to exhibit a weak protective association with prostate cancer, which has been seen in studies assessing selenium in plasma, serum, toenails, and dietary supplementation [45]. Some studies have indicated that the relationship between selenium and prostate cancer may be U-shaped, with the protective effect attenuating at concentrations above and below a specified selenium range [46]. Previous evidence has suggested that a protective association between selenium concentration and prostate cancer is more evident among smokers [11,12]. Our results support this hypothesis in our stratified analysis; we found a non-significant protective association (OR = 0.8), whereas, among participants never smoking, a non-significant increased risk (OR = 2.1) was found. Although we do not have adequate power to detect a statistically significant interaction effect, the large amount of never smokers in our study may be another explanation why an overall inverse association was not detected. However, a pooled analyses of 15 prospective studies found that selenium concentration in nails was a more reliable long-term marker (than blood) and was inversely associated with prostate cancer [47]. Similarly, a meta-analysis of 38 articles on selenium and prostate cancer found protective but heterogeneous associations for serum and plasma but a homogeneous significant protective association among three studies using nails [48].

Collection of toenails for trace element analysis in a population of men with prostate cancer and healthy controls was feasible, as the response rates for submitting the toenails samples were similar to the return of the mailed questionnaire. These data also show detectable levels of over 50% for 14 of 22 elements detected in the toenails. This included trace elements that may be important in other cancer studies, including associations seen with cancers and chromium (Cr) [49,50,51], cobalt [50,52], copper [51], iron [53], manganese [53], vanadium [51], and zinc [50,51].

Recruitment of subjects from within an existing cohort of pesticide applicators is a strength of this study, as is using the Iowa SEER registry due to the high quality of the SEER registry in identifying cancer cases. This cohort may have higher exposures to trace elements than the general population, which might be a limitation. The small sample size is a limitation. This random sample of controls and all prostate cancer cases at the time are representative of the cohort and thus should reduce the opportunity for selection bias; however, the response rate being lower than 90% could have created selection bias. While nested case-control studies should prevent selection bias from within a cohort study, this cohort of mostly farmers tends to be healthier than the general population but may have higher exposure to trace elements through chemical exposures incurred as pesticide applicators. Confounding has been addressed in the analyses, but residual confounding could exist due to inaccurate measurement of potential confounders and lack of adjustment for unknown confounders. However, currently, few risk factors for prostate cancer are well understood beyond African descent and low lycopene consumption (which is why we looked at tomato consumption). Two additional strengths of our study are using toenails as the biomarker and analyzing them using NAA. While blood and urine specimens are useful for determining short-term trace element exposure, short-term markers are rapidly cleared from the body, making them less appropriate for establishing longer-term exposure [6]. Under normal conditions, toenails grow at a rate of 1 mm per month, averaging up to 12 months for complete nail turnover [30,54]. Therefore, toenail clippings can be used as biomarkers to quantify a person’s exposure up to a year prior to their clipping. The slow growth rate of toenails permits detection of exposures over a longer time compared to blood and urine [30,54]. Since NAA offers sensitivities that are superior to those attainable by other methods, it is often considered the gold standard. Our data show a wide range of detectable levels of other long-lived trace elements that can be used in larger studies of cancer and other diseases. The detection rate for arsenic using NAA was 47% regardless of mass of the toenail sample. For selenium, the detection rate dropped from 80% (among samples ≥100 mg) to only 33% for samples with less than 100 mg, suggesting that for selenium at least 100 mg samples are needed. Detection levels of over 75% of samples were seen for aluminum, bromine, chlorine, cobalt, gold, magnesium, manganese, potassium, sodium, and zinc.

## 5. Conclusions

We found evidence of an association between higher arsenic levels in toenails and prostate cancer in a nested case-control study within the AHS. Further investigation of arsenic exposure and prostate cancer is warranted, along with further investigation into trace elements and cancer.

## Figures and Tables

**Table 1:** Self-reported characteristics of 59 prostate cancer cases and 173 healthy male controls selected within the Iowa portion of the Agricultural Health Study cohort, based on the case-control questionnaire.

Characteristic	Cases N (%)	Controls N (%)	*p*-Value ^2^
Age at pilot enrollment			
40–59	11 (19%)	46 (27%)	0.78
60–69	34 (58%)	82 (47%)	
70–79	10 (17%)	35 (20%)	
80–89	4 (7%)	10 (6%)	
Mean age, years	65.7	65.3	0.72
Married (at cohort enrollment)	57 (97%)	160 (92%)	0.27
Education at enrollment			
Less than high school graduate	1 (2%)	9 (5%)	0.44
High school graduate/GED	31 (53%)	89 (52%)	
Some college or vocational education	10 (17%)	34 (20%)	
College graduate	15 (17%)	38 (22%)	
(missing)	2 (3%)	3 (2%)	
Body Mass Index (kg/m^2^) at enrollment ^1^			
Missing	25 (42%)	47 (27%)	0.90
<25	8 (14%)	30 (17%)	
25–26.5	9 (15%)	30 (17%)	
26.6–29.9	7 (12%)	35 (20%)	
30+	10 (17%)	31 (18%)	
Smoking status			
Never	34 (58%)	93 (54%)	0.83
Current	20 (34%)	71 (41%)	
Former	5 (8%)	8 (5%)	
Mean smoking pack-years	8.5	5.9	0.23
Consumption of vegetables			
Tomatoes	54 (96%)	142 (93%)	0.34
Fresh vegetables (other than tomatoes)	31 (57%)	101 (66%)	0.28
Home grown vegetables (other than tomatoes)	9 (17%)	35 (23%)	0.35
Know they take supplements with selenium	22 (37%)	61 (35%)	0.78
Prostate cancer family history	9 (16%)	21 (12%)	0.55

^1^ From the take-home questionnaire among the cohort, not all subjects completed, creating missing values. ^2^ *p*-values excluding the missing for tests for chi-square for dichotomous factors, *t*-test for mean differences (continuous age and pack-years), and Wilcoxon rank sum test for three or more ordered categories.

**Table 2:** Case-control comparison of potential risk factors for prostate cancer in the Iowa portion of the Agricultural Health Study.

Risk Factors	Cases	Controls	Crude OR (95% CI)
Prostate Issues ^1^			
Enlarged Prostate	37/59 (63%)	45/173 (26%)	4.8 (2.6–9.3)
Prostatitis	11/59 (19%)	17/173 (10%)	1.9 (0.8–4.6)
Arsenic Exposures ^1,2^			
Lead Arsenate	3/45 (7%)	4/166 (2%)	2.9 (0.6–13.4)
Inorganic Arsenic	0/45 (0%)	0/166 (0%)	NA
Organic Arsenic	0/45 (0%)	1/166 (0.6%)	NA

OR = odds ratio; CI = confidence interval; NA= not applicable, cannot be calculated. ^1^ Prostate issues and arsenic exposures are from the take-home questionnaire among the cohort. ^2^ Inorganic arsenic and organic arsenic from self-reported exposures to arsenic from baseline cohort questionnaires.

**Table 3:** Trace elements among 59 prostate cancer cases and 173 healthy male controls selected within the Iowa portion of the Agricultural Health Study cohort.

Characteristic	Cases	Controls	Adjusted OR1 (95% CI) ^1^		Cases	Controls	Adjusted OR2 (95% CI) ^2^	
Arsenic PPM ^3^								
<0.20	7 (12%)	45 (26%)	Ref		20 (34%)	80 (46%)	Ref	
0.20–0.28	28 (47%)	57 (33%)	3.4	1.3–8.6	20 (34%)	44 (26%)	2.0	1.0–4.2
>0.28	24 41%)	71 (41%)	2.2	0.9–5.6	19 (32%)	49 (28%)	1.6	0.8–3.3
Selenium PPM ^4^								
<0.26	28 (47%)	87 (50%)	Ref		33 (56%)	101 (58%)	Ref	
0.26–0.33	9 (15%)	48 (28%)	0.6	0.3–1.4	7 (12%)	39 (23%)	0.5	0.2–1.3
>0.33	22 (37%)	38 (22%)	2.0	1.0–4.0	19 (32%)	33 (19%)	2.0	1.0–4.0

PPM = parts per million; LOD = limit of detection; Ref = reference. ^1^ Adjusted for age and smoking pack-years, where LOD replaced by LOD/sqrt(2). ^2^ Adjusted for age and smoking pack-years, where LOD replaced by the maximum likelihood estimate (MLE). ^3^ 53% below the LOD. ^4^ 28% below the LOD.

**Table 4:** Detectable rates, mean, median, and range of 22 trace elements in toenails (PPM) among 239 Iowa male subjects (66 prostate cancer cases and 173 healthy male controls).

Trace Element		All Subjects Combined				Prostate Cancer Cases			Healthy Male Controls		
	Detectable N (%)	Mean	Median	Mode	Range of Detectable Values	Detect-able N (%)	Mean	Range of Detectable Values	Detect-able N (%)	Mean	Range of Detectable Values
Aluminum (al)	239 (100%)	108.31	48.3	11.3	8.5–1683.7	66 (100%)	114.4	9.1–1347.4	173 (100%)	106.0	8.5–1683.7
Antimony (sb)	164 (69%)	0.10	0.05	0.04	0.01–3.25	44 (67%)	0.06	0.01–0.24	120 (69%)	0.12	0.01–3.25
Arsenic (as)	113 (47%)	0.29	0.2	0.2	0.1–1.8	36 (55%)	0.28	0.1–1.5	77 (45%)	0.30	0.1–1.8
Bromine (br)	239 (100%)	5.43	2.6	2	0.02–486.0	66 (100%)	11.0	1.4–486	173 (100%)	3.28	0.02–41.1
Calcium (ca)	130 (54%)	0.32	0.24	0.16	0.07–1.59	36 (55%)	0.38	0.07–1.59	94 (54%)	0.30	0.08–1.44
Chlorine (cl)	239 (100%)	0.18	0.165	0.091	0.016–0.827	66 (100%)	0.20	0.016–0.739	173 (100%)	0.18	0.06–0.827
Chromium (cr)	102 (43%)	2.50	1.75	0.6	0.4–20.9	25 (38%)	1.95	0.4–6.7	77 (45%)	2.68	0.4–20.9
Cobalt (co)	227 (95%)	0.86	0.6	0.4	0.1–23.2	64 (97%)	1.07	0.2–23.2	163 (94%)	0.78	0.1–2.7
Copper (cu)	106 (44%)	13.40	8.2	8	2.4–213.7	27 (41%)	11.2	2.4–30.9	79 (46%)	14.2	3.2–213.7
Gold (au)	189 (79%)	0.01	0.004	0.003	0.001–0.233	53 (80%)	0.01	0.001–0.09	136 (79%)	0.01	0.001–0.233
Iodine (i)	57 (24%)	61.80	2.7	0.8	0.21–2975.5	27 (41%)	3.53	0.5–8.1	30 (17%)	114.2	0.21–2975.5
Iron (fe)	66 (28%)	0.02	0.01	0.01	0.01–0.17	21 (32%)	0.02	0.01–0.05	45 (26%)	0.02	0.01–0.17
Lanthanum (la)	152 (64%)	0.21	0.095	0.07	0.02–5.44	44 (67%)	0.19	0.03–1.3	108 (62%)	0.22	0.02–5.44
Magnesium (mg)	214 (90%)	0.05	0.039	0.023	0.012–0.387	60 (91%)	0.05	0.012–0.387	154 (89%)	0.05	0.012–0.226
Manganese (mn)	236 (99%)	2.26	1.35	1	0.3–53.2	64 (97%)	2.13	0.4–10	172 (99%)	2.31	0.3–53.2
Mercury (hg)	79 (33%)	0.34	0.31	0.18	0.11–1.01	26 (39%)	0.33	0.11–0.79	53 (31%)	0.34	0.12–1.01
Potassium (k)	201 (84%)	0.14	0.119	0.058	0.013–0.687	55 (83%)	0.14	0.033–0.657	146 (84%)	0.14	0.013–0.687
Scandium (sc)	88 (37%)	0.16	0.02	0.02	0.01–6.24	30 (45%)	0.02	0.01–0.05	58 (34%)	0.22	0.01–6.24
Selenium (se)	171 (72%)	0.35	0.27	0.25	0.12–2.53	49 (74%)	0.35	0.12–1.18	122 (71%)	0.34	0.12–2.53
Sodium (na)	239 (100%)	0.14	0.1	0.06	0.01–1.52	66 (100%)	0.11	0.02–0.51	173 (100%)	0.15	0.01–1.52
Vanadium (va)	111 (46%)	0.40	0.23	0.07	0.05–4.74	34 (52%)	0.34	0.05–1.75	77 (45%)	0.42	0.06–4.74
Zinc (zn)	239 (100%)	139.00	117	83.8	0.07–1927.3	66 (100%)	145.7	42.7–779.9	173 (100%)	136.5	0.07–1927.3

N = number; PPM = parts per million.

## Data Availability

We have not archived our results due to confidentiality issues in the Agricultural Health Study and lack of requirements and support at the time of this grant. For data on this cohort, please contact AHS directly for access (https://aghealth.nih.gov/ accessed on 1 September 2024).

## Supplementary Materials

The following supporting information can be downloaded at: https://www.mdpi.com/article/10.3390/curroncol31090405/s1, Figure S1: This Flow Chart shows the recruitment numbers for cases and controls. Table S1: Comparison from the nested case-control study of 232 responders and 228 non-responders based on elements of the Agricultural Health Study Enrollment Questionnaire.
