# Supplementary material for: Trace Element Concentrations of Arsenic and Selenium in Toenails and Risk of Prostate Cancer among Pesticide Applicators
Source: Curr Oncol. 2024 Sep 14;31(9):5472–83. doi: 10.3390/curroncol31090405 (PMC11430890; doi:10.3390/curroncol31090405)
Supplement: Supplementary file 1 [file curroncol-31-00405-s001.zip › curroncol-3159320-supplementary.pdf]

Supplementary Materials: Comparison of subjects who participated vs those who did not.

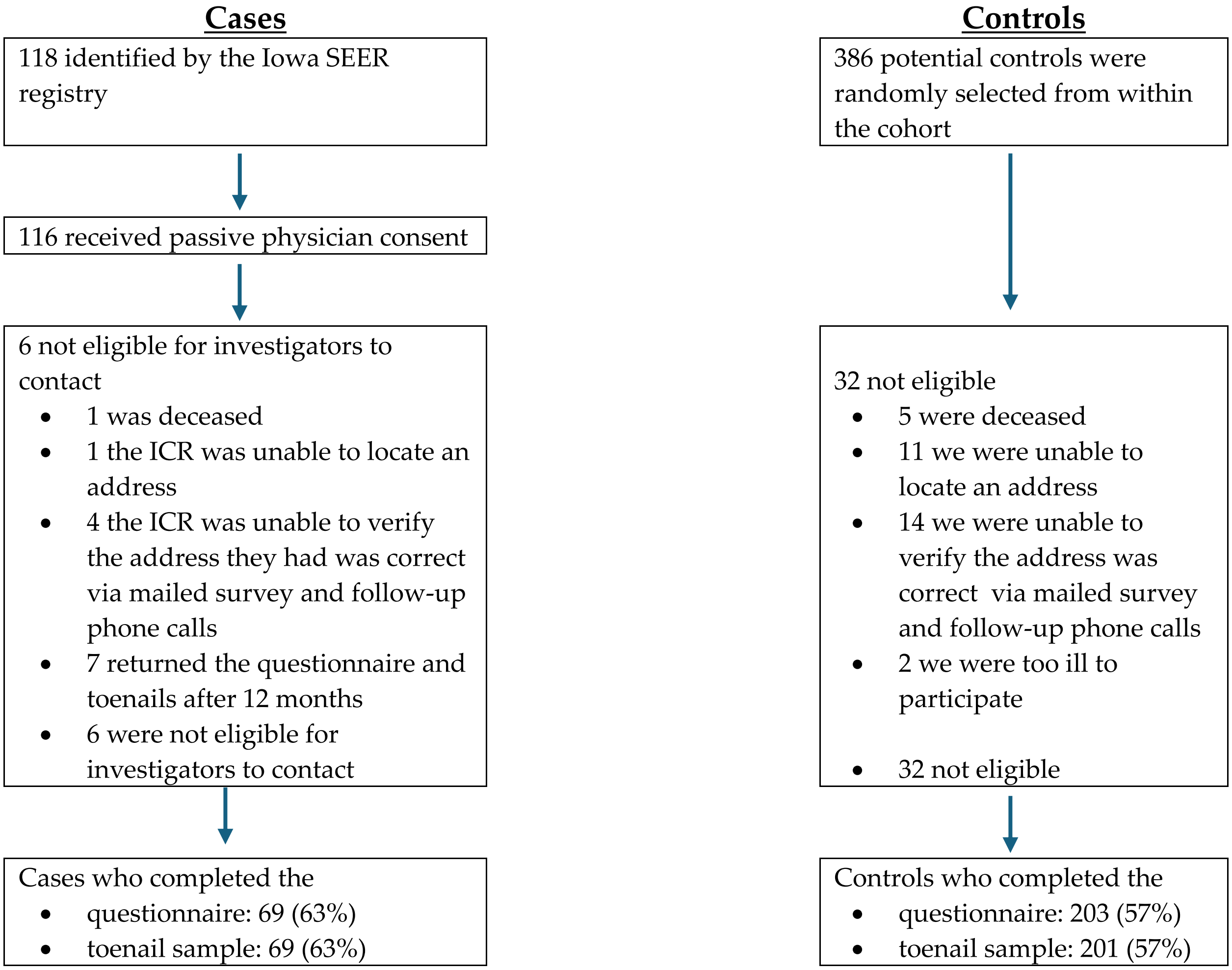

Figure S1: This Flow Chart shows the recruitment numbers for cases and controls.

Table S1: Comparison from the nested case-control study of 232 responders and 228 non-responders based on elements of the Agricultural Health Study Enrollment Questionnaire. [1]

	Non-responders N (%)	Responders (participants) N (%)	Chi-square p-value [2]
Age categories [aage]			
≤49	76 (33.3)	53 (22.8)	0.024
50-59	103 (45.2)	107 (46.1)	
60-69	41 (18.0)	55 (23.7)	
70+	8 (3.5)	17 (7.3)	
Education [aschool]			
Less than high school	14 (6.1)	10 (4.3)	0.292
High school / GED	127 (55.7)	120 (51.7)	
Some college or vocational education	46 (20.2)	44 (19.0)	
College graduate	37 (16.2)	53 (22.8)	
missing	4 (1.8)	5 (2.2)	
Race [arace]			
White	228 (100)	230 (100)	0.999
Hispanic origin [ahispan]			
Yes	1 (0.4)	2 (0.9)	0.600
No	218 (95.6)	224 (96.6)	
missing	9 (4.0)	6 (2.6)	
Eating vegetables [aveget]			
<1 per day	127 (55.7)	112 (48.3)	0.164
1 per day	39 (17.1)	48 (20.7)	
>1 per day	51 (22.4)	66 (28.4)	
missing	11 (4.8)	6 (2.6)	
Eating fruit [afruit]			
<1 per day	159 (69.7)	134 (57.8)	0.010
1 per day	36 (15.8)	55 (23.7)	
>1 per day	24 (10.5)	38 (16.4)	
missing	9 (4.0)	5 (2.2)	
Smoking during lifetime [asmok100]			
≥ 100 cigarettes	114 (50.0)	104 (44.8)	0.286
< 100 cigarettes	114 (50.0)	127 (54.7)	
missing	0 (0.0)	1 (0.4)	
Alcohol during the past 1-year [aalcfreq]			
Never	74 (32.5)	53 (22.8)	0.132
Less than one time a month	49 (21.5)	59 (25.4)	
1-3 times a month	27 (11.8)	46 (19.8)	
1 time a week	26 (11.4)	26 (11.2)	
2-4 times a week	30 (13.2)	32 (13.8)	
Almost every day	14 (6.1)	11 (4.7)	
Every day	4 (1.8)	3 (1.3)	
missing	4 (1.8)	2 (0.9)	

[1] The 232 responders included 59 cases and 173 controls, while the 228 non-responders included 46 cases and 182 controls). The Agricultural Health Study enrollment questionnaire's variable names are also provided.

[2] Chi-square test p-values are provided. The significance or not at p = 0.05 of the Cochran-Armitage Trend tests agreed with the Chi-square tests.
